# Captive environment induced greater physiological changes than probiotic treatment in *Pelophylax nigromaculatus*

**DOI:** 10.1371/journal.pone.0346236

**Published:** 2026-05-13

**Authors:** Ji-Eun Lee, Yuno Do, Jun-Kyu Park

**Affiliations:** Department of Biological Sciences, Kongju National University, Gongju, South Korea; National Museums of Kenya, KENYA

## Abstract

*Ex situ* conservation of amphibians is an effective countermeasure against various threats to populations *in situ*; however, it may entail physiological and ecological challenges that decrease individual fitness. We examined changes in the fecal bacterial community and physiological status of black-spotted pond frogs (*Pelophylax nigromaculatus*) during their transition from wild to captive environments. Additionally, we aimed to improve the physiological status and shift the fecal bacterial community through probiotic treatment. A change in fecal bacterial composition was observed after the transition from the wild to captivity and after administering probiotic treatment, whereas fecal bacterial diversity remained unchanged by environmental change and probiotics. Contrary to our expectations, changes in fecal bacterial composition did not affect innate immunity and body composition with regard to fat and lean mass and bone mineral density. Transition to the captive environment, rather than the probiotic treatment, elicited significant changes in plasma biochemical components, allowing us to predict potential issues associated with the captive environment. Our results showed that although probiotic treatment can alter the bacterial community in *P. nigromaculatus*, the specific probiotic treatment used in this study did not improve host physiology, at least in the short term. Additionally, some problems may occur even in a standard captive husbandry condition, which can be effectively monitored through physiological examination and monitoring. We suggest that a holistic approach be adopted to create captive environments informed by the ecology and physiology of the species and a thorough understanding of host-microbe interactions.

## Introduction

Amphibian species, a crucial component of global biodiversity, face unparalleled threats that have led to population decline and extinction [1]. Habitat destruction, chemical pollution, climate change, and emerging infectious diseases, such as chytridiomycosis and ranaviruses, primarily drive these crises [2,3]. The global decline in amphibians underlines the biodiversity crisis as these species play critical roles in ecosystem functionality, including nutrient cycling, predation, and prey [4]. To address this urgency, conservation efforts have incorporated multidisciplinary approaches. These include *ex situ* conservation, such as captive breeding, reintroduction, and disease mitigation, as well as conservation measures *in situ*, such as habitat preservation, restoration, and translocation [5].

*Ex situ* conservation, an essential part of biodiversity conservation, involves maintaining and breeding species outside their natural habitats, often in controlled environments, such as zoos, aquaria, and botanical gardens, or through cryopreservation techniques [6–8]. *Ex situ* conservation is an effective countermeasure against threats to populations that are difficult to conserve in natural habitats, such as large-scale epidemics or habitat loss in amphibians [9]. Amphibians are ideal candidates for *ex situ* conservation because they have low maintenance requirements and breed readily in the laboratory [9]. However, maintaining species outside their natural habitats involves challenges related to reproduction, health, and behavior. Captive conditions may lead to changes in behavior, diet composition, social structures, and pathogen exposure, thus affecting reproductive success and resistance to various metabolic diseases [10–12]. Genetic issues, such as inbreeding depression and loss of genetic diversity, may compromise the reintroduction of captive populations [13]. These challenges highlight the necessity for well-designed *ex situ* programs that ideally work in tandem with *in situ* conservation efforts. A holistic approach that includes understanding the ecological traits of species, health management strategies, such as physiological monitoring and probiotics, and population genetic considerations is likely to yield more successful conservation outcomes.

One of the major issues in *ex situ* conservation of amphibians is the constant monitoring of their health status [8]. This can be achieved through a combination of several physiological analyses [14]. Several veterinary physiological examinations, such as X-ray techniques, hematological and biochemical analyses, and immune and hormonal assays, are suitable for identifying health status and promoting environmental enrichment to improve inappropriate captive environments [11,15–17]. However, by contrast to mammals and avians, standard procedures for health monitoring are currently lacking for amphibians. This is also attributable to the absence of a reference interval for physiological parameters that facilitates the diagnosis of individual units with reference to the health status of normal individuals [17,18]. Additionally, the ecological, behavioral, and biological diversity of amphibian species, combined with the problems that may arise in monitoring their health, complicates the interpretation of captive environments and their health status [11,12]. Therefore, physiological research on various species and captive environments must be carried out steadily and consistently to monitor the health status of amphibians used for *ex situ* conservation strategies and to help maintain sustainable populations.

In addition to physiological analysis, the intricate relationship between host health and gut microbiota has gained scientific attention in recent years [19,20]. A diverse and balanced microbiome plays a pivotal role in host functions such as nutrient absorption, metabolism, and immune response modulation [21–23]. Research across diverse organisms from humans to amphibians has demonstrated the potential of microbiota-targeted interventions for disease management and health promotion [24]. In particular, probiotics, living microorganisms that confer health benefits to the host, have been extensively explored in amphibian conservation contexts. Probiotics can modify microbiota, thereby maintaining or restoring their balance [25–27]. However, their effectiveness relies on species-specific selection of beneficial microorganisms, appropriate formulation, and effective delivery methods. It is necessary to determine whether probiotic treatment can affect host physiology as well as cause changes in the microbial community in the various environments inhabited by amphibians. This has been well-studied in humans and other mammals [28–30]. Although the importance of microbial communities to host physiology has recently received attention in ectothermic animals, current studies on amphibian probiotics tend to focus fragmentarily on microbial changes or host physiology.

Therefore, in this study, we introduced captive frogs from a wild population and monitored the shift in host physiology and fecal bacterial communities during the transition from wild to captive environments. Additionally, we identified the effects of probiotic treatment, which may shift the fecal bacterial community and enhance the physiological conditions of frogs during several captive phases. We expected that the physiology and fecal bacterial communities of frogs from the wild population would gradually change during the captive period. We hypothesized that probiotic treatment would help these rearing adaptations both in terms of microbiology and physiology. Physiological analysis included veterinary clinical tests, such as hematology, X-rays, and immunoassays. Safe and highly accurate physiological tests, such as veterinary clinical examinations, are already used in taxa such as mammals and birds and have the potential to be useful for the diagnosis of amphibians [17]. Our study may improve our understanding of captive amphibian management and monitoring methods for future *ex situ* conservation strategies.

## Materials and methods

### Animal collection and breeding

The black-spotted pond frog (*Pelophylax nigromaculatus*) is a common species that is widely distributed in East Asia [31]. Because these frogs are easy to raise in the laboratory and because of their remarkable physiological response to environmental changes, they are suitable for physiological monitoring of adaptation to the captive environment [17]. We collected 12 male black-spotted pond frogs with distinct secondary sex characteristics from a rice paddy in Gongju-si, Chungcheongnam-do, during the breeding season from May to June 2022. The secondary sexual characteristics of these frogs were determined according to a nuptial pad on the first finger and the vocal sac. Female frogs exhibit large physiological variations during the breeding season. Moreover, they can breed only once per year, and population stability may suffer from collecting them. Therefore, in this study, we exclusively used stable males [17]. No frogs showed noticeable signs of body abnormalities, such as ascites, swelling, deformity, or injury. The frogs were transferred to the animal laboratory at Kongju National University and placed in plastic containers filled with clear water.

In the laboratory, the frogs were housed in individual plastic tanks with a cover (543 × 363 × 268 mm). To avoid contact with contaminants, 30 pores per inch polynomial sponge filters were provided as land mass, and fresh water was added under the sponges. A water pool was provided to supplement the water, and a hiding place was provided to reduce perceived stress. During the experiment, the photoperiod was 12/12 hours using LED lights; temperature and humidity were maintained at 23 ± 2°Cand 70%, respectively. Because there was little difference in size and weight between individuals(Mass: 31.70 ± 8.67 g; SVL: 104.95 ± 7.24 mm), we fed all frogs four mealworms (400 mg) dusted with Rep-Cal herptivite multivitamins (with beta-carotene) and Rep-Cal herptivite calcium (vitamin D3 and phosphorus 0%) once every 3 days in a feeding bowl. After confirming that the frog had completely consumed the prey items, all containers and feeding bowls were rinsed to remove fecal and other contaminants and were washed every 3 days using distilled water. Animal management and experimental procedures were approved by the Laboratory Animal Ethics Committee (KNU_2022-01) of Kongju National University.

### Experimental design

**Fig 1:**
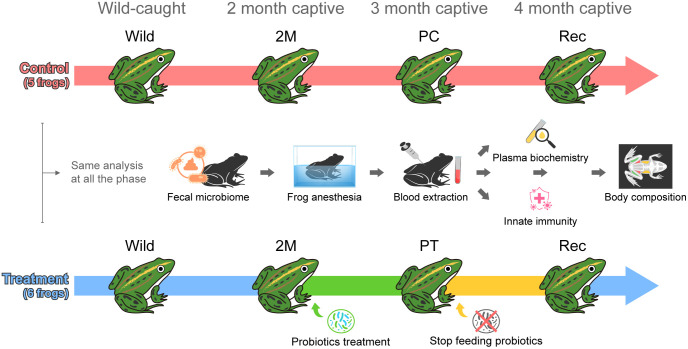
A summary of the analysis methods and experimental details used in the study. Wild-caught, black-spotted pond frogs (*Pelophylax nigromaculatus*) were raised into treatment groups, receiving probiotics, and control groups, not fed probiotics. Fecal microbiome, plasma biochemistry, innate immunity and body composition were used to monitor the shift of fecal bacterial community and the physiological change of frogs by environmental transition from wild to captivity and by probiotics.

We established two experimental conditions: six individuals in the probiotic-feeding group (treatment) and five individuals in the non-probiotic group (control). Physiological data of all frogs were collected immediately after capture in the wild. We analyzed the intestinal microbiome within 12 h of moving the frogs to the individual tanks containing sterile water and collected feces. After fecal extraction, blood was collected under anesthesia with 0.5 g/L of MS-222 (tricaine methane sulfonate). Immunological and blood chemistry analyses were performed using plasma. Finally, X-ray images were used to measure the body composition of all frogs. Detailed information on each analysis is provided in this section. We assume that 2 months of raising was sufficient for the frogs to adapt to the captive conditions. Physiological data were measured in the treatment group after probiotic supplement for 1 month to understand the changes in the physiological state and composition of the intestinal bacterial community presumed to be conveyed by probiotic treatment to breeding individuals. Lastly, physiological data were measured 1 month after the cessation of *Bacillus* supplementation to confirm whether the altered physiological or microbial state was maintained even after *Bacillus* supplementation was discontinued. During the recovery process after anesthesia, one control subject failed to regain consciousness; therefore, only five control subjects were included in the analysis. An overview of the experiments is shown as a flowchart (Fig 1). All invasive procedures, including blood sampling, were conducted under anesthesia with MS-222 to minimize potential pain and distress. At the end of the experiment, frogs were humanely euthanized by immersion in 10 g/L MS-222 solution in accordance with approved ethical guidelines.

### 2.3. Analysis of fecal bacterial community

As we needed to continuously monitor the research animals, we adopted the frog fecal microbiome analysis method and proceeded with bacterial community analysis of individuals. Using autoclaved tweezers, fecal samples were immediately transferred to a tube containing fine beads as provided with the Qiagen DNeasy PowerSoil Pro Kit (Qiagen, Hilden, Germany). To prevent cross-contamination between samples, alcohol disinfection was repeatedly performed before and after fecal sample collection. After adding lysis buffer to each fecal sample, the samples were stored at −30 °C.

Genomic DNA extraction of the fecal bacterial communities of frogs and probiotics was performed using a Qiagen DNeasy PowerSoil Pro Kit (Qiagen) according to the manufacturer’s instructions. Fecal and probiotic samples were homogenized using a TissueLyser II (Qiagen) for 5 min at a speed of 30 m/s. The concentration and quality of the extracted genomic DNA were determined using electrophoresis. PCR was performed using two primers targeting the 16s ribosomal RNA (16 rRNA) region with Illumina overhang adapter sequences, 515F and 806R. After purification using the Agencourt AMPure XP purification system (Beckman Coulter, Brea, CA, USA), the amplified PCR products were subjected to secondary index PCR using a Nextera XT Index Kit (Illumina, San Diego, CA, USA). The purified secondary PCR products were quantified using a QFX dsDNA High Sensitivity Assay Kit (Denovix Inc., Wilmington, DE, USA) and a DeNovix-QFX fluorometer (Denovix Inc.). All samples were diluted to 10 nmol/L based on the measured and pooled concentrations. Samples were sequenced using an Illumina Miniseq system (Illumina).

Paired-end sequencing data in the form of FASTQ were processed using QIIME2 software 2023.5 [32]. The DADA2 pipeline was used to perform denoising of chimera sequences, paired-end merging, quality filtering, and feature table construction [33]. We determined the trimming parameters based on Demux visualization. Taxonomic assignments were annotated and clustered using the SILVA 16S rRNA reference database (http://www.arb-silva.de; accessed July 16, 2023). The dataset contained only bacterial taxa. Alpha diversity analysis of the fecal bacterial communities was performed using the Microeco package (version 0.20.0) in R software (version 4.3.1) [34].

### Blood extraction

All frogs were anesthetized using an MS-222 (tricaine methane sulfonate) solution at 0.5 g/L in form of a bath to examine the immune function of wild frogs, and blood was extracted through cardiac venipuncture using a syringe. According to previous studies, the immune response can change within 18–24 h in response to acute stress [35], thus blood was extracted within 16 h after collecting the individuals. After blood extraction, all subjects were transferred to individual cages with oxygen-enriched clear water to ensure adequate hydration. After stabilization for 1 h, regaining consciousness was confirmed by recovery of the cornea and righting reflex [36]. Recovered frogs were immediately transferred to their tanks. The collected blood was transferred to heparinized vacuum tubes. After confirming that the blood and heparin reacted sufficiently, the blood was centrifuged at 3000 × *g* for 10 min. Thereafter, the plasma present in the supernatant was separated and stored at −30 °C until immunological and blood biochemical analysis.

### Immunological assays

We performed the bacterial killing assay as described previously [17,37]. Plasma samples (10 µL) were diluted by adding 190 µL Amphibian Ringer’s solution (ARS) at a ratio of 1:20. *Escherichia coli* (Microbio-Logics #24311-ATCC 8739, St. Cloud, MN, USA) working solution was diluted to a concentration of 10^6^ using phosphate-buffered saline. The negative control contained only 210 µL ARS, and the positive control contained 200 µL ARS and 10 µL *E. coli* working solution. Then, the 10 µL of *E. coli* working solution was added to all samples. All samples plus the negative and positive controls were incubated at 37 °C for 60 minutes. Thereafter, we added 500 µL tryptic soy broth to all samples and the controls, and then 300 µL of each was transferred to a 96-well plate in duplicate. The plates were incubated at 37 °C for 2 h, and we measured absorbance five times at 2 h intervals using a microplate spectrophotometer (wavelength 600 nm). The value of Bacterial Killing Ability (BKA) was calculated at the beginning of the bacterial Log phase using the formula 100 × [(1 − (optical density of sample/optical density of positive control)] (%). The BKA values represent the percentage of killed *E. coli* in the plasma samples compared with the positive controls.

### Blood chemistry analysis

Plasma samples extracted from the blood were used for biochemical blood analysis using an automated clinical chemistry analyzer (Hitachi Automatic Analyzer 7020, Japan). We analyzed nine biochemical components of plasma: glucose, aspartate aminotransferase (AST), alanine aminotransferase (ALT), blood urea nitrogen (BUN), creatinine, total protein (TP), albumin, triglyceride (TG), total globulin (TGB), and calcium (Ca). Glucose is an indicator of nutritional condition, osmotic pressure, metabolism, and stress in frogs [38–40]. AST levels change after damage to organs such as the heart, liver, and skeletal muscles. As ALT, which indicates hepatic condition, is too sensitive on its own, it is commonly used in combination with AST. Both components increase simultaneously, indicating hepatic function during stress [39]. BUN, a waste product of protein metabolism, and creatinine, a waste product of muscle metabolism, are used to assess renal function [41,42]. TP, TG, TGB, and albumin levels are indicators of hepatic and renal function, nutritional status, homeostasis, and blood loss [39,43]. Ca indicates the nutritional status of the kidneys [44].

### Radiography

The bone mineral density (BMD) and fat and lean body content of frogs were computed using dual-energy X-ray absorptiometry (Medikors InAlyzer, Seongnam, Korea). In general, lean body mass is calculated as the sum of muscle content and body water content [17], and body composition was measured using the same equipment. After hydrating frogs sufficiently for 2 h, we anesthetized them using MS-222 solution at 0.5 g/L in the form of a bath immediately prior to taking X-rays. In previous studies, body composition was analyzed by removing water from samples stored in 99.5% ethanol for more than 2 months [16,17]. However, in the current study, we did not adopt a method of removing body water using ethanol to continuously monitor the subjects. Considering a previous study showing that acute water intake affects body composition [45], the maximal hydration process was performed before X-ray analysis to equalize the body water content of all frogs.

### Statistical analyses

The four phases in the treatment group were termed in chronological order, i.e., wild (wild-caught frogs), 2M (breeding for 2 months), PT (probiotic treatment), and REC (recovery of initial captive condition by stopping probiotics). As the control group did not receive probiotics, we also used four phases, in chronological order, i.e., Wild, 2M, PC (period of breeding without probiotics in the control group), and REC. The physiological data and fecal bacterial community composition of the four phases for each group were analyzed. Because the purpose of this analysis was to examine temporal changes within each group, statistical comparisons were performed only among the four phases within the same group, and no direct comparisons were made between the control and treatment groups. A Kruskal-Wallis test was performed to test differences in the diversity indices (observed species, Shannon index, InvSimpson, and phylogenetic diversity [PD]) for the fecal bacterial communities of the four phases per group. After confirming the statistical differences among the groups, Dunn’s post hoc test was performed. Kruskal-Wallis tests were also used to test differences in the composition of the fecal bacterial communities of the groups at the phylum, genus, and species levels. Moreover, to test differences between groups in length and weight, BKA, plasma components (GLU, AST, ALT, BUN, CRE, TG, TP, ALB, TGB, and Ca), and body composition (fat and lean mass and BMD), we used Kruskal-Wallis and Dunn’s post hoc tests. All statistical analyses were performed using JASP software (version 0.17.3) [46], and statistical significance is reported at *p <* 0.05.

## Results

### Alpha diversity of fecal bacterial community

**Fig 2:**
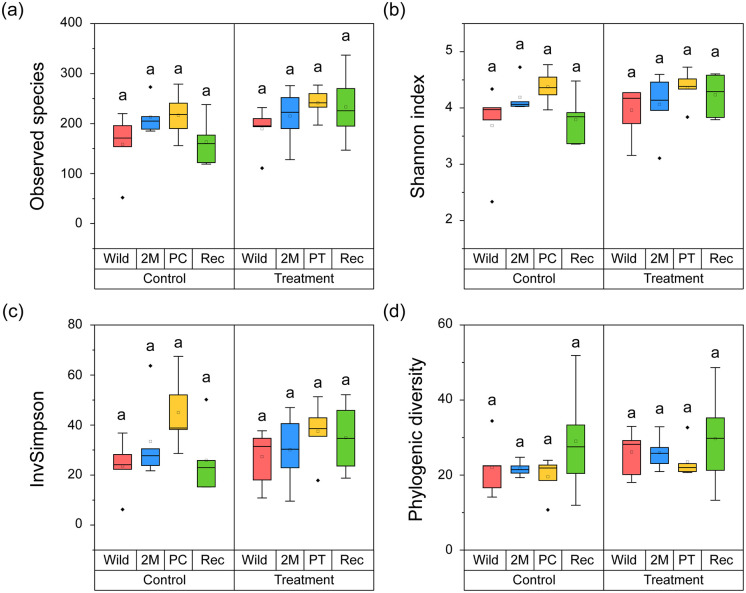
Comparison in alpha diversity of the fecal bacterial community from probiotics treatment and control group during wild to captivity: (a) Observed species, (b) Shannon index, (c) InvSimpson index, (d) Phylogenic diversity. The top and bottom of the box indicates the third and first quartiles and the ends of the both lines mean the 1.5 interquartile range. The thick line in the middle indicates the median, the dots are the outliers. The significance of differences (*p* < 0.05) was calculated from Dunn’s post hoc test after Kruskal-Wallis test, and was displayed lowercase letters.

We compared the alpha diversity of fecal bacterial communities for the four phases in each group with the observed species, Shannon, Inv Simpson, and PD indices (Fig 2a–2d). In the control group, statistically significant differences among four phases were not found in observed species (H = 4.897, *p =* 0.179), Shannon (H = 7.331, *p =* 0.062), InvSimpson (H = 7.057, *p =* 0.070), or PD (H = 1.126, *p =* 0.771) indices.

We also found no significant differences among the four phases in the treatment group for the observed species (H = 5.296, *p =* 0.151), Shannon (H = 3.553, *p =* 0.314), InvSimpson (H = 2.607, *p =* 0.456), and PD (H = 2.053, *p =* 0.561) indices. Overall, alpha diversity maintained its value in all four phases (Dunn’s test, *p >* 0.05).

### Relative abundance and functional groups of fecal bacterial communities

The fecal bacterial communities of the four phases in each group were classified at the phylum, class, and genus levels. To confirm that the fed probiotics were settled in the guts of the frogs, the relative abundance of probiotics was also determined.

**Fig 3:**
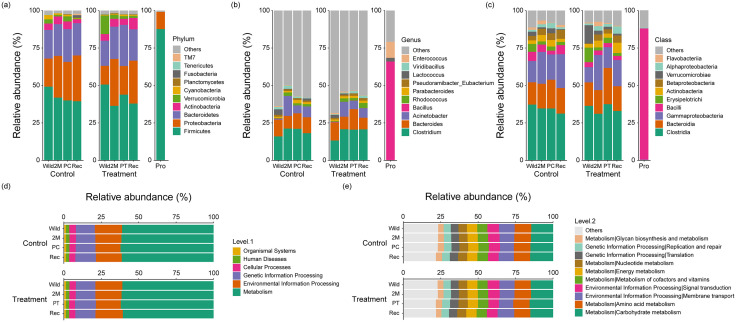
Relative abundance of fecal bacterial comparison and annotated functional groups: (a) at the phylum level from probiotics treatment and control group during wild to captivity, and from feeding probiotics (b) at the class level (c) at the genus level (d) top level of relative abundance in functional groups from treatment and control group during wild to captivity (e) second level of relative abundance in functional groups.

At the phylum level, Firmicutes showed the highest dominance throughout the experimental period in both the control and treatment groups. (Fig 3a). In the control group for the wild phase, Bacteroidetes were the second most dominant, and since the 2M phase, the abundance of Proteobacteria consistently dominated. Firmicutes in the treatment group decreased in the 2M phase (43.16%) compared to that in the wild phase (50.89%), increased slightly after feeding the probiotics (44.51%), and decreased again after the discontinuation of feeding probiotics (38.66%). Bacteroidetes showed a pattern similar to that of Firmicutes. Conversely, Proteobacteria increased during the 2M phase (29.82%), decreased during the PT phase (18.41%), and increased during the REC phase (27.90%). Among the probiotics, Firmicutes (87.63%) had the highest abundance in the two groups. Overall, captivity events altered fecal bacterial composition in both groups. The composition of the fecal bacterial community changed after feeding probiotics to the treatment group, whereas it was maintained in the control group.

At the class level, Clostridia showed the highest abundance during the entire experimental period in both the control and treatment groups (Fig 3b). Before probiotic feeding, Gammaproteobacteria showed the second highest abundance in both groups; however, after probiotic feeding, Bacteroidia increased and became dominant. However, after discontinuation of probiotic feeding, Bacteroidia decreased, and Gammaproteobacteria again had the second highest abundance. By contrast, among the probiotic treatment, Bacilli (87.63%) was the most dominant, confirming that it did not match the abundance in the control and treatment groups.

At the genus level, other genera had the highest abundances in both the control and treatment groups, followed by *Clostridium* and *Bacteroides* (Fig 3c). However, *Bacillus* (65.94%) showed the highest abundance, and discrepancies in abundance between the two groups were confirmed.

We compared the functions predicted based on the 16S ribosomal RNA gene of the bacterial communities between the control and treatment groups. At the top level, all groups had more than half of the functions of metabolism, followed by environmental information processing and genetic information processing, which had high abundance (Fig 3d). At the second level, the two groups had the highest abundance of metabolism-related carbohydrate and amino acid metabolism, followed by membrane transport and signal transduction, metabolism of cofactors and vitamins, and energy metabolism (Fig 3e). Overall, in the treatment group, metabolism-related functions increased during the PT phase, but when probiotic feeding was stopped, the original abundance was maintained. In contrast, the relative abundance of these functions was maintained in the control group.

### Physical and physiological condition affected by captive environment and probiotic treatment

**Fig 4:**
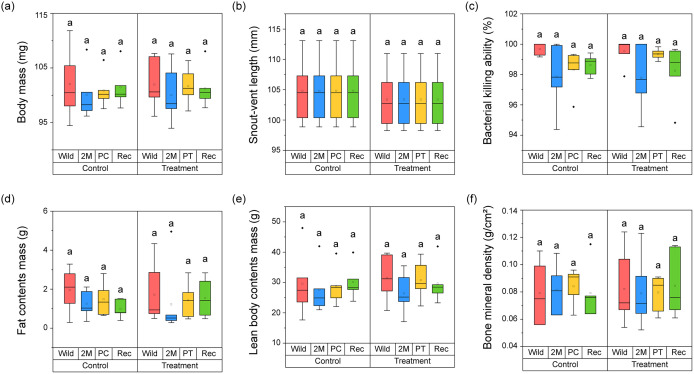
Comparison of physical condition, body compositions, and innate immunity from probiotics treatment and control group during wild to captivity: (a) body mass (b) snout-vent length indicating body length (c) bacterial killing ability indicating innate immunity (d) fat contents mass (e) lean body contents mass (f) bone mineral density. The top and bottom of the box indicates the third and first quartiles and the ends of the both lines mean the 1.5 interquartile range. The thick line in the middle indicates the median, the dots are the outliers. The significance of differences (*p* < 0.05) was calculated from Dunn’s post hoc test after Kruskal-Wallis test, and was displayed lowercase letters.

The physical condition, body composition, and innate immunity measured at each phase in the control and treatment groups were compared using Kruskal-Wallis tests (Fig 4). In the control group, there were no significant differences in body mass (H = 0.623, *p =* 0.891), snout–vent length (H = 0.000, *p =* 1.000), bacterial killing ability (H = 1.985, *p =* 0.157), fat mass (H = 1.697, *p =* 0.638), lean body mass (H = 0.977, *p =* 0.807), or BMD (H = 0.349, *p =* 0.950) in all phases.

Similar to the control group, the treatment group showed that body mass (H = 1.287, *p =* 0.732), snout–vent length (H = 0.000, *p =* 1.000), bacterial killing ability (H = 4.553, *p =* 0.208), fat mass (H = 3.327, *p =* 0.344), lean body mass (H = 2.180, *p =* 0.536), and BMD (H = 0.139, *p =* 0.987) did not change significantly during the entire experimental period. In the treatment group, the physical condition, body composition, and innate immunity of captive frogs were not affected by probiotic treatment or captivity in any of the phases (Dunn’s test, *p >* 0.05). The control group was also unaffected by captivity; their physical condition, body composition, and innate immunity were maintained from the wild to the REC phase (Dunn’s test, *p >* 0.05).

### Plasma components by captive environment and probiotic treatment

**Fig 5:**
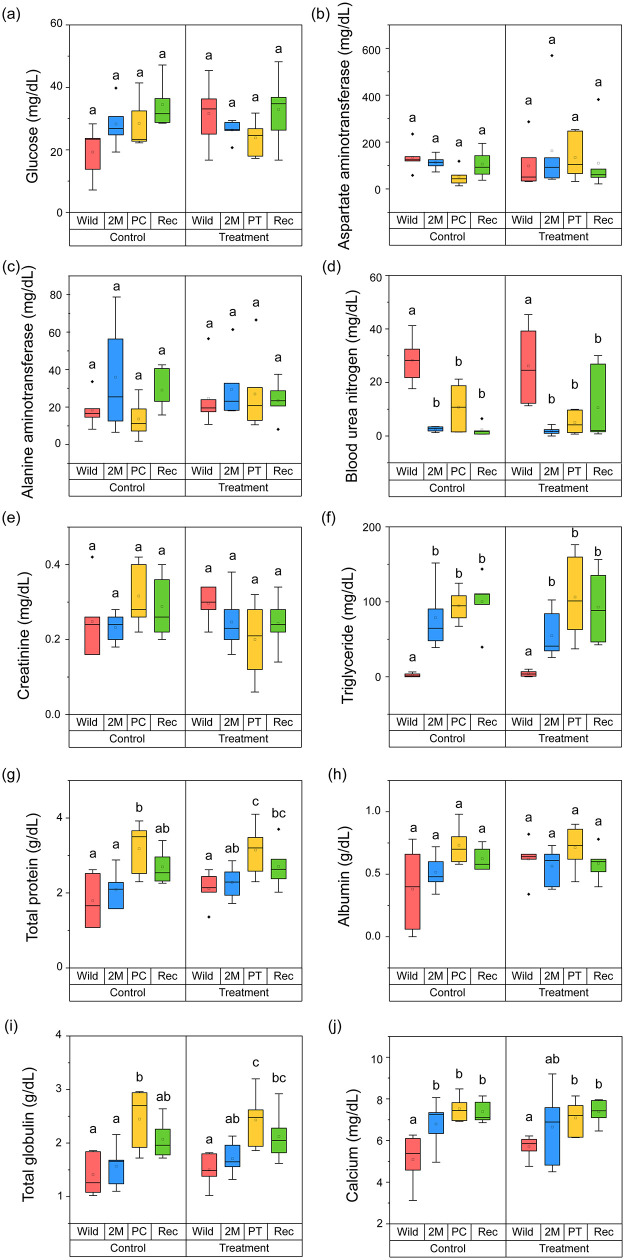
Comparison of plasma components from probiotics treatment and control group during wild to captivity: (a) glucose (b) aspartate aminotransferase (c) alanine aminotransferase (d) blood urea nitrogen (e) creatinine (f) triglyceride (g) total protein (h) albumin (i) total globulin (j) calcium. The top and bottom of the box indicates the third and first quartiles and the ends of the both lines mean the 1.5 interquartile range. The thick line in the middle indicates the median, the dots are the outliers. The significance of differences (*p* < 0.05) was calculated from Dunn’s post hoc test after Kruskal-Wallis test, and was displayed lowercase letters.

We compared the values of the plasma components in all phases between the control and treatment groups (Fig 5). In the control group, glucose (H = 6.634, *p =* 0.085), AST (H = 6.246, *p =* 0.100), ALT (H = 3.914, *p =* 0.271), creatinine (H = 2.961, *p =* 0.398), and albumin (H = 4.743, *p =* 0.192) levels were not significantly different across phases. Similarly, in the treatment group, there were no significant differences in the glucose (H = 4.067, *p =* 0.254), AST (H = 1.347, *p =* 0.718), ALT (H = 0.767, *p =* 0.857), creatinine (H = 4.597, *p =* 0.204), or albumin (H = 3.725, *p =* 0.293) in all phases.

In contrast, BUN (H = 11.251, *p =* 0.010), TG (H = 11.683, *p =* 0.009), and Ca (H = 9.789, *p =* 0.020) levels significantly changed after captivity for 2 months, compared with the control groups. BUN rapidly decreased (Dunn’s test, *p <* 0.05) during 2 months under captive conditions and was maintained (Dunn’s test, *p >* 0.05) steadily thereafter. In contrast, TG and Ca levels increased (Dunn’s test, *p <* 0.05) in the 2 months of captivity, and were similarly maintained (Dunn’s test, *p >* 0.05) steadily thereafter. TP (H = 7.838, *p =* 0.049) and TGB (H = 9.227, *p =* 0.026) increased after 3 months (in PC) and slightly decreased after 4 months of captive conditions (REC in control; Dunn’s test, *p <* 0.05).

In the treatment group, BUN (H = 10.947, *p =* 0.012), TG (H = 15.020, *p =* 0.002), TP (H = 8.923, *p* = 0.030), TGB (H = 12.924, *p* = 0.005), and calcium (H = 8.782, *p* = 0.032) levels were similar to those in the control. BUN rapidly decreased (Dunn’s test, *p <* 0.05) after 2 months of captivity and then remained steady (Dunn’s test, *p >* 0.05), whereas TG and Ca gradually increased (Dunn’s test, *p <* 0.05) after 2–3 months of captivity and then remained steady (Dunn’s test, *p >* 0.05). TP and TGB increased and peaked (Dunn’s test, *p <* 0.05) during probiotic treatment and then slightly decreased again, but similar responses were observed in the control results, indicating that probiotic treatment was not causing the effect.

## Discussion

### Probiotic treatment and host physiology

**Fig 6:**
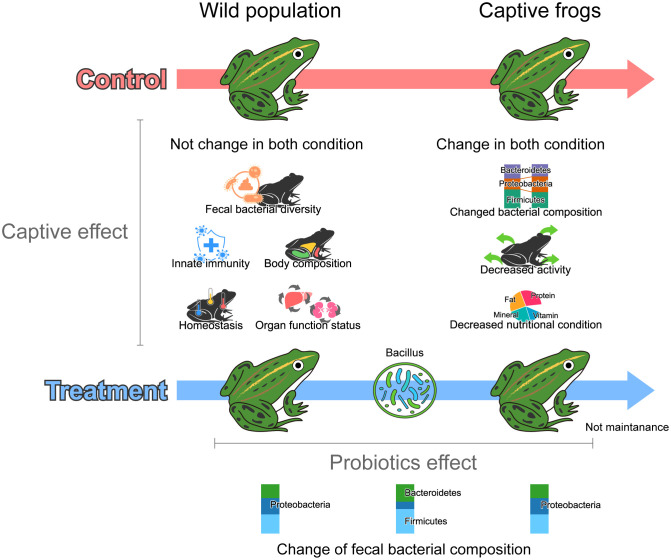
The fecal bacterial composition temporarily changed during probiotics treatment, but innate immunity, body composition, and alpha diversity of fecal bacteria did not change. However, the altered bacterial structure was not maintained after removal of the probiotics treatment, whereas physiological changes were observed in both treatment and control groups in captivity. These physiological changes, therefore, show that probiotics treatment does not provide significant support in terms of the physiological state of the host during the transition from wild to captivity.

In this study, we examined the physiological status of wild-derived captive frogs and determined whether probiotic treatment of the gut microbiota improved their physiological and health conditions (Fig 6). Probiotic treatments are generally expected to induce positive physiological responses in animals; they have been used on the skin and gut microbiome of frogs to alleviate diseases and stress [25,26,47]. In our study, no significant effects of probiotic treatment were observed on the gut microbial diversity or functional groups.

Nevertheless, probiotic treatment altered the composition of the fecal bacterial community. During the probiotic treatment phase, frogs had a higher abundance of Firmicutes and Bacteroidetes and a lower abundance of Proteobacteria at the phylum level. However, there was no increase in *Bacillus*, which belongs to Firmicutes, as observed at the class and genus levels. This suggests that while probiotic treatment changed the microbial composition, this was likely the result of microbial interactions rather than direct colonization, which altered the fecal bacterial composition [27]. This colonization appeared to be transient because the fecal bacterial composition was not maintained when the probiotic treatment was discontinued. In contrast, Firmicutes aid in carbohydrate fermentation, nutrient efficiency, and absorption [48], and Bacteroidetes play a role in food digestion, gut homeostasis, and immune regulation through polysaccharide breakdown, immune system development, and maintenance of the gut mucosa [49]. These two bacterial groups are also symbiotic, and their relative proportions, especially the higher proportion of Firmicutes, mediate increased caloric intake efficiency and energy storage [50,51]. Therefore, we expected that these changes in fecal bacterial composition caused by probiotic treatment would help improve the physiological state of frogs during captivity. However, the observed physiological responses may reflect multiple interacting factors, including microbial shifts and temporal effects associated with captivity, making it difficult to attribute these changes solely to probiotic treatment.

Contrary to our expectations, the probiotic treatment did not show significant support in terms of host physiology; all physiological components showed a similar pattern of change in all phases in the control and treatment groups. There were no changes in the immunity or body composition of the frogs, and analysis of blood plasma biochemistry showed no significant changes in glucose, AST, ALT, or albumin levels, which are related to hepatic function [52,53]. Several previous studies have attempted to alleviate disease or health conditions in frogs treated with probiotics, but no significant effects have been found [25,47,54]. It is possible that our experiment was too short to expect a probiotic effect or that we did not find an effect because we used adult frogs that were nearly complete in growth and did not show any signs of disease. In addition, the relatively small sample size used in this study may have limited the statistical power to detect subtle physiological changes associated with probiotic treatment. Therefore, the absence of significant physiological effects observed in this study suggests that no clear changes were detected under the conditions tested, although smaller effects may not have been fully captured. Future studies including a larger number of individuals will be necessary to more clearly evaluate the physiological responses to probiotic treatment. These results show that probiotic treatment of the gut microbiota, even if it results in a microbial response, may not always be noticeable; it may not be noticeable in the short term or in normal frogs. Future studies on the effects of probiotic treatment over longer periods or in relation to growth and disease may produce different results.

### Shift in host physiology in captive environmental transitions

In contrast, we detected physiological changes under captive conditions rather than under probiotic treatment. A decrease in BUN and an increase in Ca, TG, and TGB in the blood were observed during the same phase in both the control and treatment groups. In general, simultaneous changes in BUN, TP, creatinine, and Ca levels are indicative of renal dysfunction [42], and simultaneous changes in glucose and proteins in the blood are indicative of changes in homeostatic function [55,56]. However, we concluded that the changes in these blood components were not indicative of deterioration in renal function or changes in homeostasis. In essence, BUN and creatinine levels do not increase simultaneously, which is unlikely to indicate a renal abnormality [42,57]. By contrast, the changes in TP in our results were driven by TGB rather than albumin; homeostatic changes are typically associated with changes in albumin [58,59] and in our study, albumin continued to maintain non-significant differences in the blood.

We interpret this as a physiological response to nutritional imbalance and reduced activity during captivity. Decreased BUN levels can indicate renal dysfunction or decreased protein metabolism; however, it can also indicate malnutrition [60,61]. Similarly, an increase in TGB can be caused by inflammatory diseases but can also indicate malnutrition [55,62]. The simultaneous decrease in BUN and increase in TGB appears to be the result of relatively poor nutritional condition during captivity adaptation of frogs compared to the wild population. Indeed, frogs in captivity frequently experience poor nutritional status due to a narrower prey spectrum than those in the wild [10,63], which has been an ongoing concern in the management of captive amphibians [44], and the appropriate use of calcium or vitamin supplements has been recommended to address this [40]. We alternated between mealworms and crickets and consistently dusted them with the calcium and vitamin supplements commonly used in amphibian captivity; however, this may not sufficiently cover the wide spectrum of prey in the wild [44]. This nutritional imbalance can manifest as reduced appetite, lowered immunity, and health abnormalities such as obesity or reduced muscle mass [44]. Fortunately, this did not lead to changes in immunity or body composition in our study; nevertheless, we believe that this could lead to health-related problems if maintained over the long term. However, previous studies involving captive amphibians did not identify the detailed mechanisms underlying this process. Long-term monitoring of these physiological changes during husbandry is necessary. Although we interpreted these changes primarily as responses to nutritional imbalance and reduced activity in captivity, alternative mechanisms cannot be excluded. In particular, shifts in the gut microbial community may influence host metabolic processes, including lipid metabolism, even in the absence of detectable immune responses [64]. Previous animal studies have demonstrated that gut microbiota can alter host lipid metabolism and circulating triglyceride levels [65]. Future studies integrating metabolomic or functional microbiome analyses would help clarify these potential pathways [66].

Increased Ca in the blood can be caused by an increase in Ca or vitamin D intake, but it can also be caused by a decrease in activity; in a state of reduced activity, the weight load on the bones is reduced and Ca is released from the bones into the blood [ 67,68]. This can also lead to changes in TG; an increase in TG can indicate both a lack of exercise or nutritional imbalance and is also associated with the diagnosis of overweight and obesity [52,55]. Prior to the experiment, we determined that the cages were of sufficient environment to accommodate the frogs based on general amphibian husbandry recommendations. The captive conditions were designed following these recommendations, including the provision of water access and refuge structures to reflect the semi-aquatic ecology of this species [69]. However, the locomotor performance of this species may have been overlooked. Black-spotted pond frogs are semi-aquatic frogs that use water and land alternately and are excellent jumpers and swimmers [31,70]. Much more focus has been placed on the locomotor performance of hind limbs than in other species because these frogs do not have defensive mechanisms, such as tasteless substances or camouflage coloration, to avoid predators [70]. Given the strong locomotor ability of this species, the cage size used in this study may still have limited the activity levels of the frogs [11]. By contrast, arboreal frogs such as tree frogs, walkers such as toads, and burrowers may not require as large a breeding space as black-spotted pond frogs and may exhibit different responses [71]. This indicates that cages of different sizes should be considered depending on the locomotory type of the amphibian. It also suggests that ecological traits, such as the locomotory type of the species, should not be overlooked in captivity and that further physiological monitoring of these effects should be performed.

## Conclusion

This specific probiotic treatment temporarily altered the composition of the fecal bacterial community in *P. nigromaculatus*, however, the altered bacterial composition was not maintained after the probiotic treatment was discontinued, and the treatment did not positively influence the physiological changes associated with the transition from wild to captive environments. Even under standard captive husbandry conditions, overlooked factors may still occur and can be detected through physiological analyses. However, the lack of deterioration in body composition and immunity suggests that these physiological changes do not pose significant problems in captivity and require long-term monitoring. We suggest that ecological understanding of this species should not be overlooked when creating captive amphibian husbandry environments. Our results are expected to improve the understanding of the monitoring of captive amphibians and their interactions through probiotic treatment in *ex situ* conservation strategies.
